# Supplementary material for: Pressure-Enhanced Photocurrent in One-Dimensional SbSI via Lone-Pair Electron Reconfiguration
Source: Materials (Basel). 2022 May 27;15(11):3845. doi: 10.3390/ma15113845 (PMC9182005; doi:10.3390/ma15113845)
Supplement: Supplementary file 1 [file materials-15-03845-s001.zip › materials-1701786-supplementary.pdf]

Supporting Information

Pressure-Enhanced Photocurrent in One-dimensional SbSI via Lone-Pair Electrons Reconfiguration

Tianbiao Liu,[1] Kejun Bu,[1*] Qian Zhang,[1, 2] Peijie Zhang,[1] Songhao Guo,[1] Jiayuan Liang,[1] Bihan Wang,[1] Haiyan Zheng,[1] Yonggang Wang,[1] Wenge Yang,[1] and Xujie Lü[1*]

[1] Center for High Pressure Science and Technology Advanced Research, Shanghai 201203, P. R. China

[2] College of Material and Chemical Engineering, Zhongyuan University of Technology, Zhengzhou 450007, P. R. China

Figures and tables

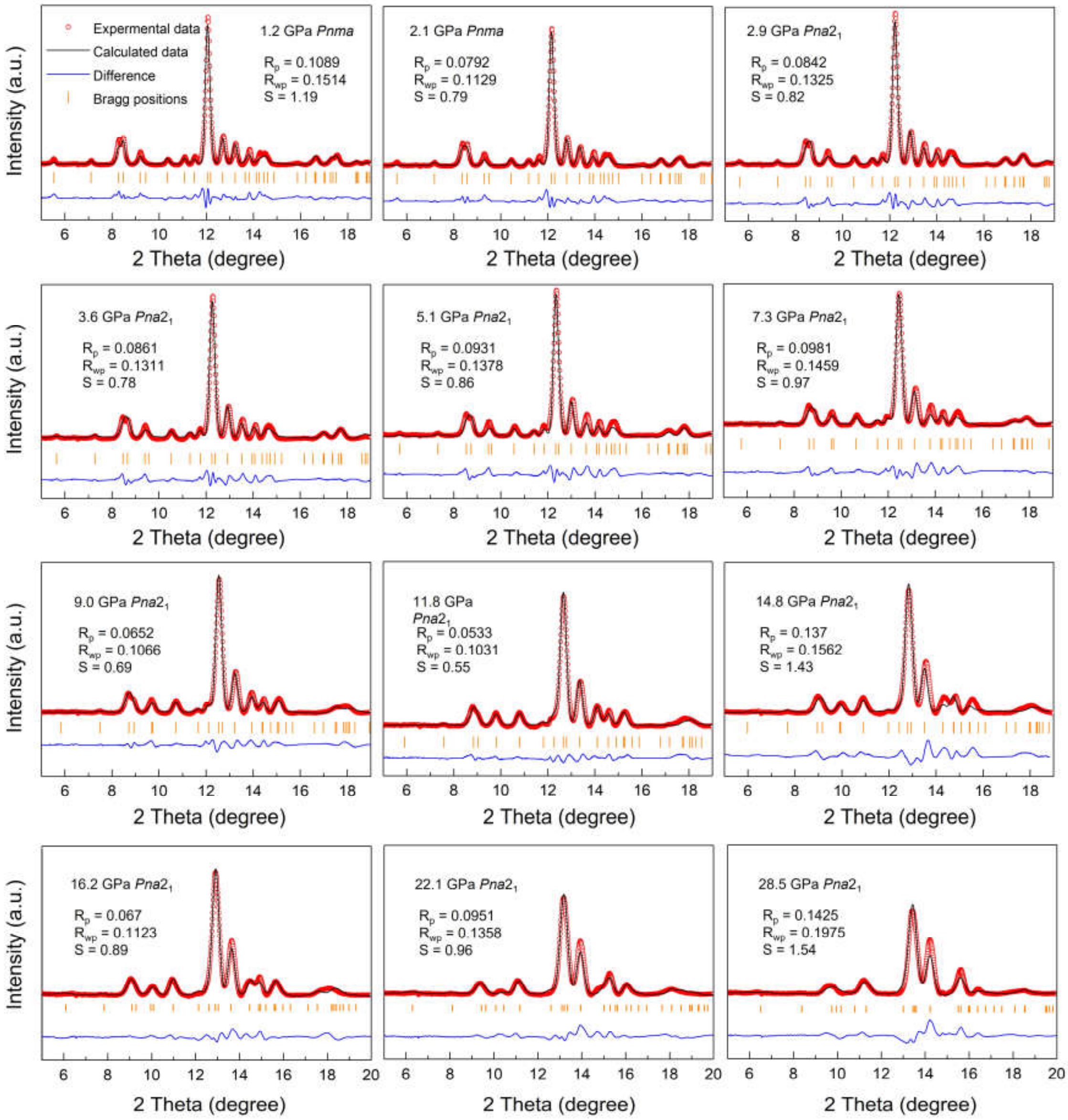

Figure S1. Rietveld refinement results of SbSI at selected pressures.

Table S1. The lattice parameters of the SbSI at selected pressures.

Pressure (GPa)	Space group	*a* (Å)	*b* (Å)	*c* (Å)	*V* (Å^3)
1.2	*Pnma*	8.3869	9.9900	4.0707	341.064
2.1	*Pnma*	8.3036	9.8979	4.0409	332.112
2.9	$Pna2_1$	8.2484	9.8287	4.0298	326.702
3.6	$Pna2_1$	8.2094	9.7728	4.0216	322.648
5.1	$Pna2_1$	8.1399	9.6954	4.0040	315.994

7.3	$Pna2_1$	8.0485	9.5781	3.9802	306.831
9.0	$Pna2_1$	7.9601	9.5120	3.9547	299.435
11.8	$Pna2_1$	7.8664	9.3614	3.9326	289.598
13.2	$Pna2_1$	7.8303	9.3421	3.9170	286.534
14.8	$Pna2_1$	7.73860	9.2300	3.8756	276.828
16.2	$Pna2_1$	7.68650	9.1520	3.8598	271.524
18.8	$Pna2_1$	7.5750	8.9960	3.8520	262.494
22.1	$Pna2_1$	7.4870	8.8990	3.8078	253.701
24.2	$Pna2_1$	7.4390	8.7990	3.769	246.702

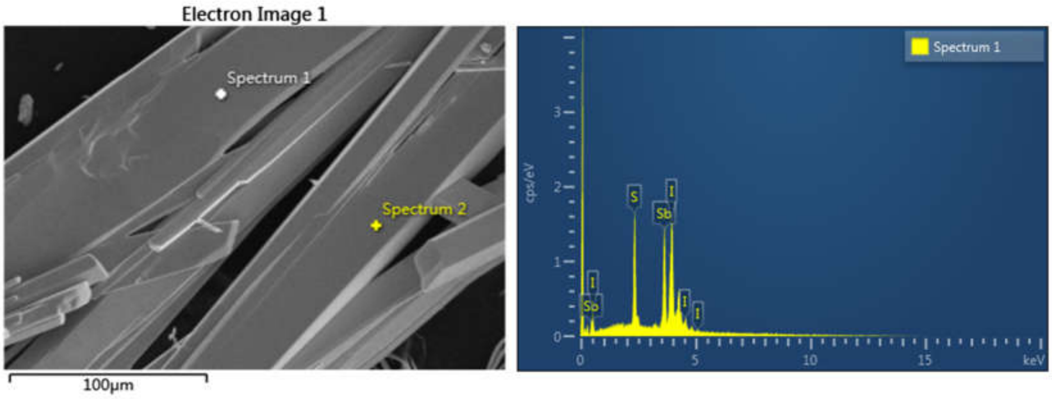

Figure S2. The scanning electron microscope (SEM) image and X-ray energy dispersive spectroscopy (EDS) of SbSI single crystal.

Table S2. Energy-dispersive X-ray analysis results of SbSI.

Element	Wt%	Atomic %
S	10.77	31.85
Sb	45.74	35.64
I	43.50	32.51
Total:	100.00	100.00

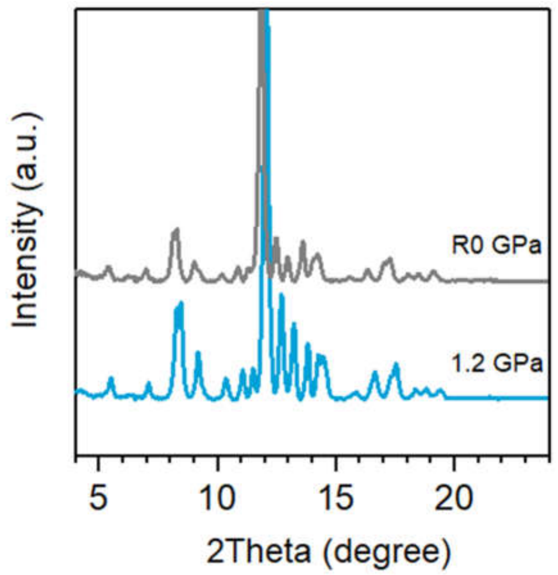

Figure S3. XRD patterns of the SbSI initial sample and the recovered sample.

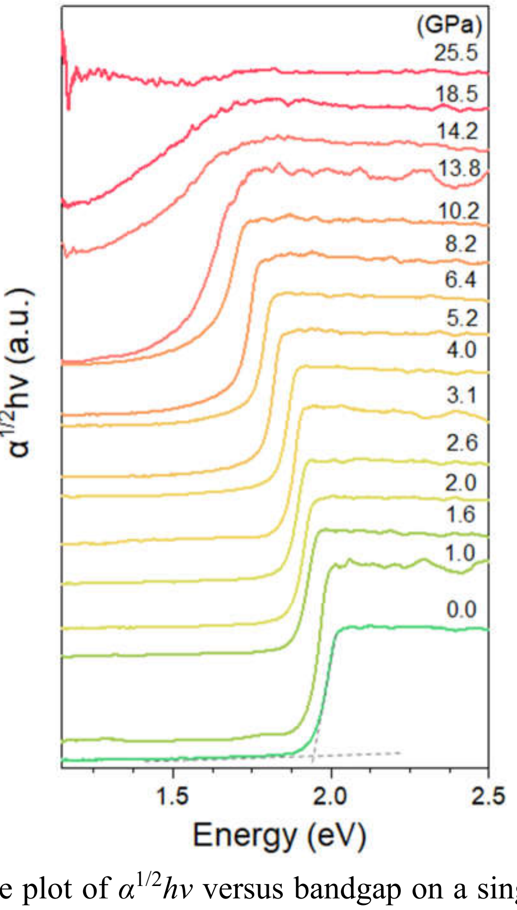

Figure S4. Selective the plot of $\alpha^{1/2}hv$ versus bandgap on a single-crystal SbSI under compression.

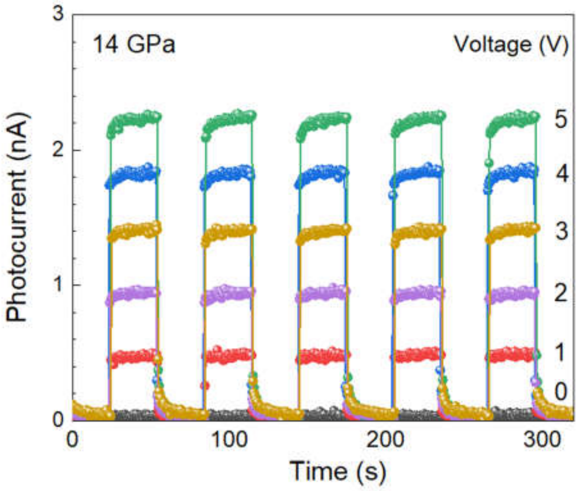

Figure S5. Photocurrent of 14 GPa at different bias voltages.

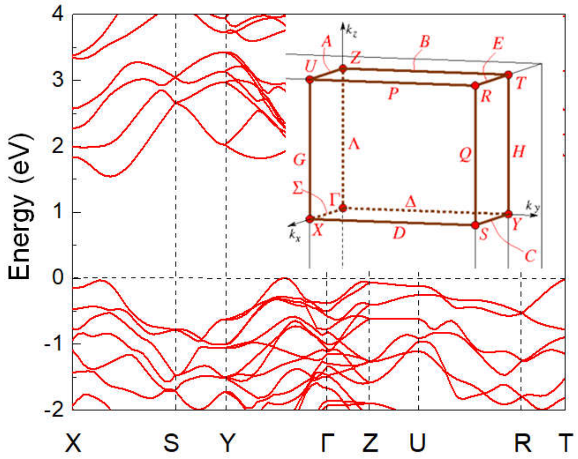

Figure S6. Electronic band structure of SbSI.
